# An Evaluation of Drug Prescribing Patterns and Prescription Completeness

**DOI:** 10.3390/healthcare12222221

**Published:** 2024-11-07

**Authors:** Saadeldin Ahmed Idris, Tarig Mahmoud Ahmed Hussien, Faraj Farih Al-Shammari, Hatim Adam Nagi, Abdelhafiz Ibrahim Bashir, Gamal Eldin Mohamed Osman Elhussein, Rania Abdeen Hussain Abdalla, Halima Mustafa Elagib Mohammed, Wafa Elhassan Abdelaziz, Amal Daher Alshammari, Hend Faleh Hamad Alreshidi, Hind Naif Mhaileb Alshammari, Somaia Ibrahim Bashir Ibrahim

**Affiliations:** 1 Department of Surgery, College of Medicine, University of Hai’l, Hai’l 55476, Saudi Arabia; 2 Department of Ob/Gyne, College of Medicine, University of Hai’l, Hai’l 55476, Saudi Arabia; tm.ahmed@uoh.edu.sa (T.M.A.H.); r.hussain@uoh.edu.sa (R.A.H.A.); 3 Department of Pharmacy, University Medical Clinics, University of Hai’l, Hai’l 55476, Saudi Arabia; ghar_911@hotmail.com; 4 Department of Family and Community Medicine, College of Medicine, University of Hai’l, Hai’l 55476, Saudi Arabia; ha.adam@uoh.edu.sa (H.A.N.); amal.alshammari@uoh.edu.sa (A.D.A.); hendfal@hotmail.com (H.F.H.A.); hindnaif09@gmail.com (H.N.M.A.); 5 Department of Physiology, College of Medicine, University of Hai’l, Hai’l 55476, Saudi Arabia; ah.bashir@uoh.edu.sa; 6 Department of Pediatrics, College of Medicine, University of Hai’l, Hai’l 55476, Saudi Arabia; g.osman@uoh.edu.sa (G.E.M.O.E.); somaiabashir@yahoo.com (S.I.B.I.); 7 Department of Pharmacology, College of Medicine, University of Hai’l, Hai’l 55476, Saudi Arabia; h.elagib@uoh.edu.sa; 8 Department of Medicine, Ha’il General Hospital, General Directorate of Health Affairs on Hai’l Region, Hai’l 55422, Saudi Arabia; weelhassan@moh.gov.sa

**Keywords:** constant dose, polypharmacy, prescribing patterns, prescription completeness, rational usage of drugs, suitability

## Abstract

**Background/Objectives:** The rational use of medicines, in accordance with the World Health Organization (WHO) guidelines, is crucial for optimizing healthcare outcomes. This cross-sectional study aimed to evaluate drug prescribing patterns and assess prescription completeness based on the WHO core drug use criteria. A comprehensive analysis was conducted at the University Clinic in the Northern region, Kingdom of Saudi Arabia (KSA). **Methods:** The study assessed drug prescribing patterns and examined prescription completeness by analyzing various parameters recommended by the WHO core drug use criteria. **Results:** Upon analyzing the 615 prescriptions, it was observed that each prescription had a mean of 2.56 prescribed drugs. Multiple medicines per prescription were prevalent in 71.4%, whereas polypharmacy was evident in 2.9%. Analgesics were the most frequently prescribed medication, accounting for 50.4% of the prescriptions, followed by supplements (31.7%), decongestants (16.1%), cough syrup (12%), and antihypertensive and diabetes treatments at 17%. Furthermore, antimicrobials were prescribed in 21.5% of the prescriptions. During the evaluation, it was found that 19.8% of the prescriptions were incomplete, lacking important information on dosing, duration, and drugs not suited to the diagnosis. **Conclusions:** Most of the parameters evaluated in this study were determined to fall outside the range of recommended guidelines criticized by the WHO. As a result, the implementation of efficient intervention programs, such as education initiatives, is recommended to enhance the practice of rational drug use. **Contribution:** This study highlights the importance of improving prescription indicators at the national level, focusing on both medication prescribing characteristics and prescription quality as a practice.

## 1. Introduction

The provision of healthcare services relies heavily on the use of medications. According to the World Health Organization (WHO), rational drug use involves ensuring that patients receive the most effective treatment at a reasonable cost, with suitable dosages, and for an appropriate duration [1]. Communication between the prescriber and dispenser typically takes place through a written instruction known as a prescription [2]. The prescription functions as an official record generated by a doctor to guide pharmacists or nurses caring for the patient. In the event of any misconstruction, there is a risk of administering incorrect treatment, exacerbating the patient’s condition, posing potential health risks, and imposing a greater financial burden on health services. Prescription faults and errors play a significant role in contributing to medication errors [3].

Prescription errors refer to mistakes that occur during the prescription writing process, which can involve drug selection, dose, route of treatment, and treatment duration. These errors can be classified into two primary types: errors of omission, when there is some incompleteness in the prescription; and errors of commission, when incorrect information is included. Furthermore, prescribing faults encompass illogical, incongruous, and ineffective prescribing, as well as under- or overprescribing, which results from imprecise diagnosis or choices related to treatment or monitoring. Errors in dispensing medication can be attributed to inaccuracies, inadequate clarity in writing, or deficient prescriptions [3,4]. By gaining a comprehensive perception and exploring prescription patterns, noble information can be gleaned regarding the ever-changing perspective of healthcare and the utilization of medicines [5]. Effectively managing irrational prescribing practices requires a thorough evaluation of prescribing patterns and event rates. In addition, prescription research plays a crucial role in drug epidemiology as it affords valuable insights into the extent and nature of drug exposure. This kind of research aids in developing a comprehensive understanding of medication usage, which in turn supports more effective regulation of prescribing practices [6]. The WHO identifies several key components within a prescription. These elements encompass assessing the merit, range, and tendencies in medicine consumption, as well as drug usage practices, including non-commercial brands, constant dosage, and medications listed in the National List of Essential Medicines. Moreover, prescription research involves assessing adherence to prevailing nationwide strategies and examining the comprehensiveness of prescriptions in the means of dosage, preparation, extent, frequency, and other pertinent factors [7].

Numerous studies have assessed prescription patterns [1,2,3,5,7,8], yielding varied outcomes. Some investigations reported deviations from standard prescribing practices [1,2,7,8], while others indicated a commendable prescribing pattern [5]. Notably, Navadia et al. found that 82% of prescriptions were legible [3].

In Saudi Arabia, drug prescribing involves licensed healthcare professionals assessing patient needs, selecting appropriate medications, and following strict regulations and guidelines to ensure safety and effectiveness in treatment. This study sought to evaluate drug prescribing patterns and examine the completeness of prescriptions by analyzing a range of parameters outlined in the WHO core drug use criteria.

## 2. Materials and Methods

### 2.1. Study Design

A cross-sectional study of prescriptions was carried out at the University Medical Clinics (UMCs) in the Northern region of KSA, which has one outpatient pharmacy and one pharmaceutical store within UMCs. The study included an extensive multiple analysis of prescriptions of patients who attended the UMCs from December 2023 to February 2024.

### 2.2. Study Population and Sampling Strategy

The study included all electronic prescriptions that met certain inclusion criteria in the specified period. Such criteria included prescriptions from outpatient clinics in UMCs with patients aged 18 or more who came for first-time visits or returned for reassessment after their initial visit. Nevertheless, some prescriptions were eliminated under defined exclusion criteria. These encompassed prescriptions for those who required management in an emergency room or were less than 18 years of age.

### 2.3. Sample Size

The sample size was calculated and determined in accordance with WHO recommendations, which specify an optimal minimum of 600 prescriptions for evaluation [8]. The prescriptions were randomly selected using computer-generated numbers from the outpatient pharmacy.

### 2.4. Data Collection

Prescriptions were gathered across multiple departments, including Internal Medicine, General Surgery, Obstetrics and Gynecology, Dermatology, Ophthalmology, Otolaryngology, Family Medicine, and General Practice. Data were gathered using a pre-designed questionnaire that included patient information such as age and gender, diagnosis and treatment details including the number and type of medications, their generic or commercial names, duration, and dosage. This information was then manually entered into a computer. The operational definitions were as follows:

Errors: A prescription was classified as erroneous if any of the following components were absent in the patient’s prescription order: patient’s name, age, gender, weight, file number, and diagnosis; medicinal information including polypharmacy, generic name, dose, frequency, and duration; and prescriber’s information including physician’s name, rank, specialty, date, and signature [9].

Essential Drug List: Refers to a compilation of medications that are deemed essential or necessary to meet the healthcare requirements of a specific population. In this study, the term “Essential Drug List” was used interchangeably with the term “essential medicines list” to refer to the same concept.

Rational use of drugs: Refers to prescribing the appropriate medication, at the correct dosage and strength, to the right patient, for an adequate duration of time, and diagnosis. Each prescription was evaluated by four expert independent physicians. This concept emphasizes the importance of ensuring that medications are used judiciously and in a manner that maximizes their effectiveness while minimizing potential risks or adverse effects.

Antibiotics: Antibiotics are compounds that are either naturally produced by certain fungi, bacteria, and other organisms or derived from them. These substances have the ability to kill or inhibit the growth of other microorganisms, such as bacteria, by targeting specific cellular processes or structures. Antibiotics are widely used in medical practice to treat bacterial infections and have played a crucial role in combating various infectious diseases.

Polypharmacy: Refers to the circumstance in which five or more medications are prescribed.

Multiple medications: Refers to the situation in which two to four medications are prescribed.

Generic name: Refers specifically to the International Non-proprietary Name (INN) of a drug. This is the official and universally recognized name assigned to a medication based on its active pharmaceutical ingredient (API).

### 2.5. Data Analysis

The data were explored to evaluate the prescription patterns within UMCs, employing the prescription guidelines recommended by the WHO. These guidelines encompassed various aspects, including the average number of drugs for each prescription, the fraction of medicines prescribed utilizing generic terms, the proportion of prescriptions containing antibiotics, the proportion of prescriptions involving injectable medicines, and the proportion of drugs aligned to the essential drugs list. The data collected were initially organized in an Excel sheet and subsequently imported into SPSS for analysis. Categorical data were expressed as frequencies and percentages. The numerical data were analyzed using descriptive methods and expressed as mean and standard deviation.

### 2.6. Ethics

This study was conducted in accordance with ethical guidelines. Since the data were collected from existing pharmacy records and involved no direct interaction with patients, there was no requirement for informed consent. The anonymity and confidentiality of the patients’ information were maintained throughout the study to ensure compliance with ethical standards. Measures included de-identifying data, restricting access to sensitive information, and securely storing records to prevent unauthorized disclosure.

## 3. Results

### 3.1. Sample Characteristics

The study included 615 prescriptions randomly extracted from the pharmacy. The majority were issued to females, constituting 61.1% (n = 376) of all patients with a female-to-male ratio of 1.6:1. Their mean age was 38.27 ± 14.1 years (range, 18 to 78 years). Fewer than half of them were younger patients aged less than 35 years (41.8%), as shown in Table 1.

**Figure 1:**
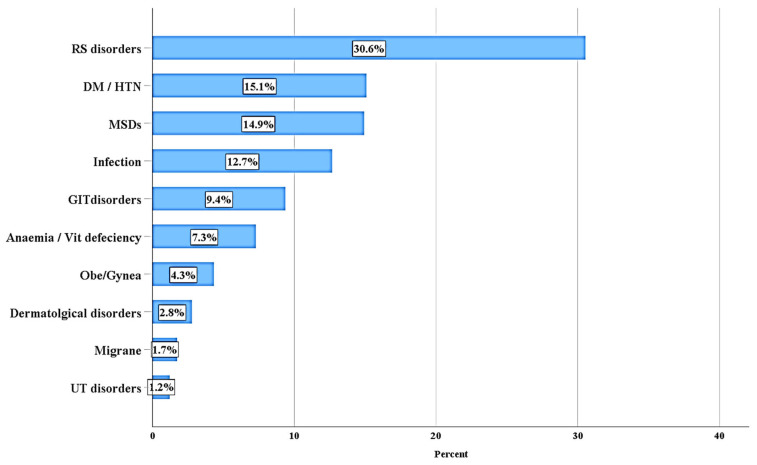
Illustrates the diagnosis of patients who were administered medication.

This study examined a wide variety of medical conditions within the prescriptions scrutinized. Respiratory disorders (RSs) were the most common diagnoses, accounting for 29.8% of the cases. The next most prevalent diagnoses were DM/HTN (diabetes mellitus/hypertension) and MSDs (musculoskeletal disorders), which made up 15.6% and 15.4% of the prescriptions, respectively (Figure 1).

A total count of 1573 drugs were extracted from prescriptions, with a mean of 2.56 drugs per prescription (SD = 1.4). The predominant brand products prescribed were analgesics, antibiotics, supplements (such as Multivitamins, Vitamin D, and Iron), and decongestants (Table 2 and Table 3).

**Figure 2:**
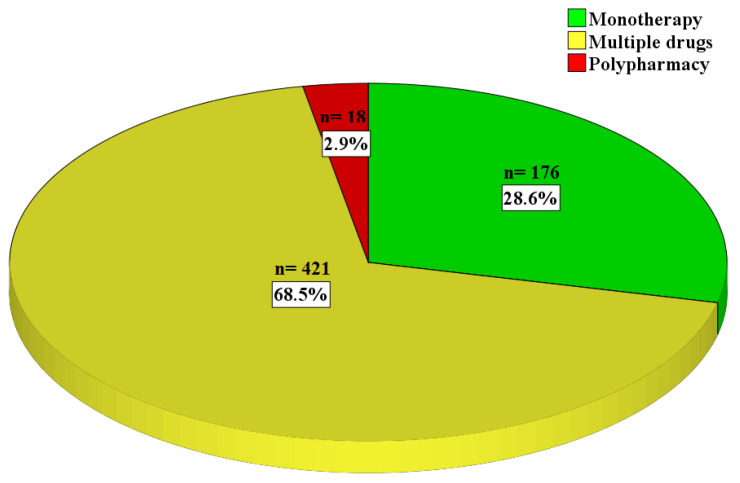
The mode of prescribing pattern (n = 615).

Out of all the prescriptions, 439 (71.4%) consisted of multiple medicines per prescription; within those 18 prescriptions (2.9%), polypharmacy contained more than 5 drugs (Figure 2). The mean count of analgesics per prescription was 0.7 ± 0.8 counts.

All medicines were written in prescriptions using their generic names, which are consistent with the Essential Medicines List of Saudi Arabia. Nevertheless, there were a few instances when the prescriptions did not conform to World Health Organization (WHO) recommendations, leading to a prevalence rate of 19.3% (n = 119). Still, a small number of prescriptions, about 6.3% (n = 39), failed to indicate the dosage. Moreover, very minimal prescription rates such as only 2% (n = 12) did not take into account the given period they should be taken for.

### 3.2. Completeness of Prescription

To evaluate the completeness of prescriptions, various factors related to patients, treatments, and prescribers were taken into consideration. Specifically, each information within the prescription was assessed for its inclusion. The study revealed that the full name, gender, and age of the patients were filled in all prescriptions. However, the patient’s weight was missing in 99.2% of the prescriptions. On the other hand, the dosage, frequency, and duration of the prescribed drugs were filled in 93.7%, 93.2%, and 98% of the prescriptions, respectively (Table 4). Overall, it was determined that 19.8% of the prescriptions were incomplete, omitting one or more pieces of information.

#### 3.2.1. Indicators of Prescribing

Within the context of the 615 prescriptions that were examined, a cumulative total of 1573 drug products were prescribed. On average, each prescription contained 2.56 drugs. It is noteworthy that all drugs were prescribed using their generic names. Antibiotics were prescribed in 132 encounters, accounting for 21.5% of the total. The three most frequently prescribed antibiotics were macrolides (45 instances, 34.1%), penicillin (25 instances, 18.9%), and metronidazole (21 instances, 15.9%), as shown in Table 5.

#### 3.2.2. Service-Peculiar Indicators

Within the clinic’s pharmacy, an essential drug list is maintained, consisting of 336 drugs. However, during the study period, only 56 of these drugs (16.7%) were available, as indicated in Table 6.

## 4. Discussion

Hospital pharmacy facilities must prioritize the efficient and effective delivery of pharmaceutical care to guarantee that patients receive the utmost available quality of care. Evaluating prescription patterns in the health-providing setting is a comprehensive process that involves the collaboration of the prescriber, the patient, and the pharmacist [10].

Most of the patients (66.1%) were female, while less than half (41.8%) were young and below the age of 35. The mean age of the patients was 38.27 years. Similar results were reported by Al-Harajin et al. in Al-Ahsa, Saudi Arabia [11].

Amidst the multitude of studies conducted on medicinal errors in Saudi Arabia and the Middle East, substantial variation exists in the definition of these errors. Few studies concentrate on the existence or lack of prescription items, while others scrutinize the accuracy of information or explore the occurrence of adverse drug reactions [1,3,4,6,7,9,12,13,14,15].

The specific prescribing indicators used can vary depending on the healthcare system, guidelines, and available resources in a particular setting [16].

The application of different standards, including those established by the WHO, FDA, and British National Formulary, to measure errors introduced complexity due to variations in the assessment criteria among them [1,3,4,6,7,9,11,12,15,17].

The prescription order plays a vital role in the interaction between doctors and patients, underscoring the significance of legibility, accuracy, and completeness to minimize errors in medication dispensing and administration. To ensure the prescription is comprehensive, prescribers must complete all the specified parameters on the prescription. These parameters encompass patient info (detailed name, gender, age, weight, file number), medication info (generic designation, dose, frequency, length of usage), and doctors’ info (name, rank, signature, and date as well as the time of prescription). For optimal practice, prescribers should ensure that all necessary information (such as patient, medication, and doctors’ details) is recorded on the prescription [6,18].

The study revealed that there were no errors in recording variables such as the patient’s name, age, sex, and diagnosis, as well as the doctor’s name, rank, signature, and date. This error-free outcome can be attributed to the utilization of computer software. The findings contradict the reports of other researchers [4,6,9].

By incorporating the patient’s weight, the pharmacist can assess the suitability of the prescribed dose, allowing for verification of its appropriateness [19]. The current study revealed that a mere 0.8% of prescriptions contained the patient’s weight, resulting in a significant absence rate of 99.2%. This finding aligns with similar studies conducted in Saudi Arabia (96.8%) [17], Libya (95.2%) [19], and Ethiopia (99.6%) [6]. Interestingly, other studies also indicated that physicians consistently failed to mention the patient’s weight [20,21].

Nevertheless, the completeness of the remaining patient-related details and prescribers’ information had been documented with accuracy and exhibited a completeness rate of 100%.

In terms of medicinal info, it was observed that all medications were prescribed using generic names, which is inconsistent with a study conducted by Ansari and Neupane in Nepal [4], where they reported that 83% of medications were prescribed using brand names. The utilization of generic names when prescribing medications offers numerous advantages for hospital pharmacies. Firstly, it enhances inventory control by reducing the variety of drug names, which facilitates more efficient management of drug procurement on a contractual basis. Additionally, prescribing generic drugs helps minimize confusion among pharmacists and is often a more cost-effective alternative to branded medications. In Saudi Arabia, the utilization of generic medications in prescribing has increasingly established itself as a widespread practice [12]. Conversely, when medications are prescribed by brand name, it may signify the implementation of aggressive promotional tactics by pharmaceutical companies [18]. Furthermore, the completeness rates for drug frequency, dosage, and duration were relatively low, measuring 93.2, 93.7, and 98%, respectively. These are significantly lower when compared to those reported in other studies [4,14,17].

The doctors’ info had been recorded optimally, achieving a completeness rate of 100%. The current study demonstrated that UMCs exhibited comparatively higher standards of practice in comparison to similar studies conducted by others [1,3,4,6,7,8,9,12,13,14,15,18].

On the other hand, overprescription refers to the excessive prescribing of multiple drugs beyond what is clinically necessary. This practice has been linked to various negative consequences, including drug–drug interactions, an elevated risk of adverse drug events (ADEs) resulting from combined or synergistic effects, noncompliance with medication, deteriorating patients’ health, raised mortality rates, and elevated health-system expenditures [18]. In the current study, it was observed that the average number of drugs per prescription was 2.56, indicating a tendency towards overprescribing at our clinics in comparison to the WHO’s recommended limit of less than 2 [22]. This finding emphasizes the presence of multiple medicinal prescriptions in our setting. This was similar to the findings by others [18,23,24,25], but it was comparable to other studies when monotherapy was the most common treatment approach [5,26].

The prevalence of antibiotic resistance is escalating in healthcare settings as well as the community. The level of resistance within a population is closely associated with the percentage of patients taking antibiotics and the extent of their exposure to antimicrobials. Augmented usage contributes to the development of resistance [6]. The ideal antibiotic prescription rate recommended by the WHO falls within the range of 20% to 26.8% [27]. In the present study, the percentage of prescriptions involving antibiotics was determined to be 21.5%, which is comparatively lower than the percentages reported in other studies, where it ranged between 38% and 58.6% [6,18].

In the present study, macrolides emerged as the most predominantly prescribed antibiotics, comprising 34.1% of prescriptions. This observation is consistent with prior research, which suggests that broad-spectrum agents, especially fluoroquinolones and macrolides, are the antibiotics most frequently utilized for adults in ambulatory care settings [28]. The pronounced utilization of macrolides in the current study may be attributable to the elevated resistance rates to penicillin observed in Saudi Arabia [29], coupled with the efficacy of macrolides in treating respiratory tract infections [30], which represent the predominant diagnoses in this research.

The availability of medicine in the pharmacy at UMCs was unacceptably low at 16.7%. This figure has been significantly impacted by the government’s transition to an expanded electronic service, which is aimed at drastically improving health services and ensuring the availability of medications. This involves connecting governmental health services with public pharmacies to make it more convenient for patients to collect treatments from the nearby public pharmacy. In general, most prescription information parameters in the current study exhibited a higher level of adherence and completeness.

### 4.1. Strengths and Limitations

The study’s strengths include a comprehensive evaluation of drug prescribing patterns within a large sample size of 615 prescriptions, enhancing the reliability of the findings. It adhered to some extent to WHO guidelines, providing a framework that could be used for comparison to assess the completeness of prescriptions and rational use of medicines in the future. Additionally, the study highlights significant insights into multiple medications and polypharmacy prevalence, as well as prescription errors, which are critical for improving healthcare practices.

On the other hand, the study exclusively concentrated on the UMCs located in the Northern region, KSA; as a result, the findings may not reflect prescription practices in different healthcare settings or regions. Moreover, there is a potential for selection bias since the study may have included a specific group of patients or a limited time frame, which might not accurately represent the overall prescription patterns within the clinics. Additionally, the study’s utilization of a cross-sectional design provides a snapshot of prescription practices at a particular moment, and conducting longitudinal studies would offer a more comprehensive understanding of prescription patterns.

### 4.2. Implications for Practice

The findings of this study underscore the need for enhanced training and education for healthcare providers on rational drug prescribing. Implementing targeted interventions, such as workshops and continuing medical education, can improve adherence to WHO prescribing guidelines. Furthermore, addressing the high prevalence of multiple medications through multidisciplinary approaches, involving pharmacists in medication management, may reduce associated risks and improve patient outcomes. Establishing standardized prescription protocols and checklists can enhance completeness and accuracy in prescribing. Finally, ongoing surveillance and evaluation of prescribing practices are essential to identify trends and areas for improvement, ultimately fostering safer and more effective medication use.

## 5. Conclusions

In this study, a notable level of adherence to the Saudi essential drug list is noted, with a majority of prescriptions being written using generic names. Moreover, the prescribing of antibiotics fell within the optimal range recommended by the WHO; however, it was relatively low in comparison to health facilities in other countries. These findings suggest a positive trend towards appropriate prescribing practices, including the use of essential drugs and generic names and cautious antibiotic prescribing. However, certain deficiencies in prescription practices were identified, necessitating improvement. The primary errors identified in this study revolve around the absence of essential information, such as dosage, frequency, duration of treatment, overprescription, and patient weight.

The study revealed that the average number of medications per prescription exceeded the recommended thresholds, indicating its inappropriate prevalence. To effectively address this, interventions involving an interprofessional approach, which often includes the expertise of a clinical pharmacist, have proven to be most successful. Considering that UMCs primarily manage chronic diseases as well as no emergency conditions, it is not uncommon for prescriptions to contain a higher number of medications. To improve prescribers’ adherence to fundamental prescription writing practices, it is advisable to offer in-service training.

## Figures and Tables

**Table 1:** The characteristics of the patients included in the study (n = 615).

Characteristics	Mean	SD
Age	38.27	14.1
	**Frequency**	**Percent**
Age groups/year		
18–25	180	29.3
26–35	77	12.5
36–45	147	23.9
46–55	143	23.3
>55	68	11.1
Gender		
Male	239	38.9
Female	376	61.1

**Table 2:** Number of prescriptions that contain a particular medicine (n = 615).

Medicine	Frequency	Percentage
Analgesics	310	50.4
Supplements (Vitamins, minerals, and herbal)	195	31.7
Antibiotics	132	21.5
Decongestant	99	16.1
Cough syrup	74	12.0
DM medications	60	9.8
Antacid	49	8.0
HTN medications	44	7.2
Others	273	44.4

**Table 3:** Number of drugs prescribed per prescription (n = 615).

Number of Drugs	Frequency	Total Drugs	Percent
Monotherapy	176	176	28.6
2 items	159	318	25.9
3 items	124	372	20.2
4 items	100	400	16.3
5 items	38	190	6.2
6 items	12	72	2.0
7 items	3	21	0.5
8 items	3	24	0.5
Total	615	1573	100.0

**Table 4:** Results of an evaluation conducted at UMCs to assess the completeness of prescriptions (n = 615).

Patient Info		Medicinal Info		Prescriber’s Info	
Consideration	n (%)	Consideration	n (%)	Consideration	n (%)
Full name	615 (100)	Generic name	615 (100)	Full name	615 (100)
Gender	615 (100)	Dose	576 (93.7)	Rank	615 (100)
Age	615 (100)	Frequency	573 (93.2)	Specialty	615 (100)
Weight	5 (0.8)	Duration	603 (98)	Date	615 (100)
Diagnosis	615 (100)			Signature	615 (100)
File No.	615 (100)				

**Table 5:** Antibiotic prescription pattern (n = 132).

Antibiotic	Frequency	Percent
Macrolides	45	34.1
Penicillin	25	18.9
Metronidazole	21	15.9
Cephalosporin	16	12.1
Aminoglycosides	14	10.6
Others	11	8.3
Total	132	100.0

**Table 6:** Service-peculiar indicators at UMCs.

Service-Peculiar Indicators	Availabilities in the Medical Clinics	Circulated to the Doctors
Existence of a copy of EDL or formulary	Existed	Yes
% existence of the medication in the store	16.7%	No

## Data Availability

The data that support the findings of this study are available from the corresponding author upon reasonable request. Due to privacy and ethical considerations, the data will not be publicly shared.

## Abbreviations

ADEs: Adverse Drug Events, API: Active Pharmaceutical Ingredient, Derm: Dermatology, DM: Diabetes Miletus, EDL: Essential Drug List, GIT: Gastrointestinal Tract, HTN: Hypertension, INN: International Non-proprietary Name, MSDs: Musculoskeletal Disorders, Obe/Gynea: Obstetrics and Gynecology, RSs: Respiratory Disorders, SPSS: Statistical Package for the Social Sciences, UMCs: University Medical Clinics, UT: Urinary Tract, WHO: World Health Organization.
